# RETRACTION: The 12‐15‐Lipoxygenase is a Modulator of Alzheimer's‐Related Tau Pathology *In Vivo*

**DOI:** 10.1111/acel.70524

**Published:** 2026-04-29

## Abstract

**RETRACTION**: GiannopoulosP. F. , JoshiY. B. , ChuJ. , and PraticòD. , “The 12‐15‐Lipoxygenase is a Modulator of Alzheimer's‐Related Tau Pathology *In Vivo*
,” Aging Cell 12, no. 6 (2013): 1082–1090, 10.1111/acel.12136.23862663 PMC3838477

The above article, published online on 17 July 2013 in Wiley Online Library (wileyonlinelibrary.com), has been retracted by agreement between the journal Editor‐in‐Chief, Monty Montano; The Anatomical Society; and John Wiley & Sons Ltd. The retraction has been agreed upon following an investigation into concerns raised by the corresponding author, D. Praticò. This identified image duplication of the tau1 bands in Figures 3A and 5A. As a result, the data and the conclusions are considered unreliable.

D. Praticò and Y. B. Joshi agreed to the decision to retract. P. F. Giannopoulos and J. Chu were informed of the decision to retract but remained unresponsive.

**RETRACTION**: P. F. Giannopoulos , Y. B. Joshi , J. Chu , and D. Praticò , “The 12‐15‐Lipoxygenase is a Modulator of Alzheimer's‐Related Tau Pathology *In Vivo*
,” Aging Cell 12, no. 6 (2013): 1082–1090, 10.1111/acel.12136.23862663 PMC3838477

The above article, published online on 17 July 2013 in Wiley Online Library (wileyonlinelibrary.com), has been retracted by agreement between the journal Editor‐in‐Chief, Monty Montano; The Anatomical Society; and John Wiley & Sons Ltd. The retraction has been agreed upon following an investigation into concerns raised by the corresponding author, D. Praticò. This identified image duplication of the tau1 bands in Figures 3A and 5A. As a result, the data and the conclusions are considered unreliable.

D. Praticò and Y. B. Joshi agreed to the decision to retract. P. F. Giannopoulos and J. Chu were informed of the decision to retract but remained unresponsive.

